# Increased Expression of RhoA in Epithelium and Smooth Muscle of Obese Mouse Models: Implications for Isoprenoid Control of Airway Smooth Muscle and Fibroblasts

**DOI:** 10.1155/2013/740973

**Published:** 2013-06-11

**Authors:** Kristie R. Ross, Rebecca J. Darrah, Craig A. Hodges, LaTresa Lang, Thomas J. Kelley

**Affiliations:** ^1^Departments of Pediatrics, Case Western Reserve University and Rainbow Babies and Children's Hospital, 10900 Euclid Avenue, Cleveland, OH 44106-4948, USA; ^2^Frances Payne Bolton School of Nursing, Case Western Reserve University, 8th Floor BRB, 10900 Euclid Avenue, Cleveland, OH 44106-4948, USA; ^3^Department of Genetics and Genome Sciences, Case Western Reserve University, 10900 Euclid Avenue, Cleveland, OH 44106-4948, USA; ^4^Department of Pharmacology, Case Western Reserve University, 10900 Euclid Avenue, Cleveland, OH 44106-4948, USA

## Abstract

The simultaneous rise in the prevalence of asthma and obesity has prompted epidemiologic studies that establish obesity as a risk factor for asthma. The alterations in cell signaling that explain this link are not well understood and warrant investigation so that therapies that target this asthma phenotype can be developed. We identified a significant increase in expression of the small GTPase RhoA in nasal epithelial cells and tracheal smooth muscle cells from leptin-deficient (ob/ob) mice compared to their wild-type counterparts. Since RhoA function is dependent on isoprenoid modification, we sought to determine the role of isoprenoid-mediated signaling in regulating the viability and proliferation of human airway smooth muscle cells (ASM) and normal human lung fibroblasts (NHLF). Inhibiting isoprenoid signaling with mevastatin significantly decreased the viability of ASM and NHLF. This inhibition was reversed by geranylgeranyl pyrophosphate (GGPP), but not farnesyl pyrophosphate (FPP), suggesting specificity to the Rho GTPases. Conversely, increasing isoprenoid synthesis significantly increased ASM proliferation and RhoA protein expression. RhoA expression is inherently increased in airway tissue from ob/ob mice, and obesity-entrained alterations in this pathway may make it a novel therapeutic target for treating airway disease in the obese population.

## 1. Introduction

The prevalence of both obesity and asthma has increased dramatically in the past two decades [1–3]; over 35% of adults and 17% of children in the USA are categorized as obese [2], and the prevalence of asthma in children under 14 years increased more than 150% between 1980 and 1999 [3]. While numerous epidemiological studies have shown that obesity is associated with the development of asthma [4–8], the alterations in cell signaling induced by obesity that might lead to airway disease are not well understood. Understanding the pathophysiology of airway disease in the growing population of obese asthmatic children is critical to the development of novel therapies specific to this phenotype. Obesity has been shown to influence a number of signaling pathways involved in inflammation and airway hyperresponsiveness [9–14]. 

Airway responsiveness can be impacted by remodeling of the airways, a process considered to be irreversible. Although the study by Aaron et al. only provides indirect evidence of a relationship between obesity and airway smooth muscle remodeling, it is consistent with the relationship between obesity and vascular remodeling. The increase in airway smooth muscle mass is likely secondary to both hyperplasia and hypertrophy [13, 14]. Mathematical modeling studies suggest that increased smooth muscle mass in itself contributes to the airway hyperresponsiveness that characterizes asthma [15]. Corticosteroids, the mainstay of therapy for asthma, are known to affect the contractility and proliferation of airway smooth muscle [16]. However, there is evidence that asthmatic airway smooth muscle exhibits alterations in cell signaling that reduce its sensitivity to glucocorticoids [17]. Furthering our understanding of the signaling mechanisms that regulate airway smooth muscle growth is crucial to the development of novel therapies that prevent or reverse structural airway changes. 

**Figure 1:**
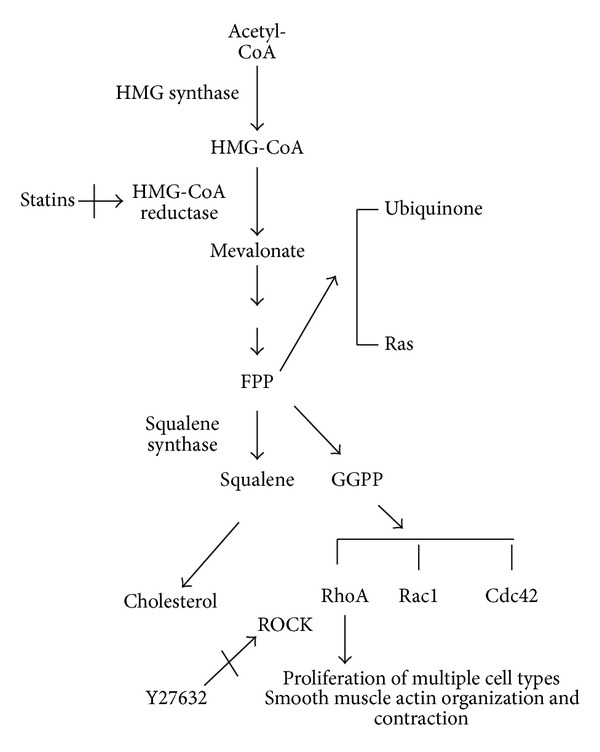
The cholesterol synthesis pathway. A branch point off the cholesterol synthesis pathway results in the synthesis of the isoprenoids farnesyl pyrophosphate (FPP) and geranylgeranyl pyrophosphate (GGPP). FPP and GGPP serve as important lipid attachments for a variety of proteins. Geranylgeranylation is necessary for the function of the Rho family of GTPases, including RhoA.

Identifying a pathway that can account for obesity-related inflammation and smooth muscle regulation would lead to a better understanding of the impact of obesity on cell signaling and identify new areas of possible therapeutic intervention. Our previous work has identified an increase in the expression and activation of the small GTPase RhoA in cells and tissues from both cystic fibrosis (CF) and Niemann-Pick type C (NPC) [18–20]. Both of these diseases are characterized in part by perinuclear accumulation of free cholesterol and upregulation of the isoprenoid/cholesterol synthesis pathway [21–24]. Isoprenoids are produced within the cholesterol synthesis pathway and are used to modify a number of signaling proteins (Figure 1). RhoA activation is modified by covalent addition of the 20-carbon isoprenoid group geranylgeranyl pyrophosphate [25]. We have identified in CF models the impact of RhoA and the related contributions of the isoprenoid pathway in inflammation and the control of TGF-β1 signaling [18, 23]. We have also demonstrated specifically in NPC fibroblasts the impact of the isoprenoid pathway on NPC cell viability and proliferation [20]. Given our previous findings, we hypothesized that RhoA expression would be increased in airway tissues from obese mouse models and that isoprenoid signaling would exhibit control of fibroblast and smooth muscle cell viability and proliferation. 

Previous studies supporting the hypothesis that obesity alters isoprenoid signaling include work demonstrating that mice lacking the obesity gene product leptin (ob/ob) exhibit increased expression of the isoprenoid synthesis enzyme geranylgeranyl diphosphate synthase (GGPS) compared to controls [26]. In another study examining the impact of obesity on hyperinsulinemic responses, Goalstone et al. found that ob/ob mouse liver, aorta, and skeletal muscle contained elevated content of Ras GTPase, a protein that also requires modification by isoprenoids [27]. If isoprenoid-mediated signaling is important in regulating airway smooth muscle proliferation, obesity-induced alterations in this pathway would be hypothesized to contribute to airway remodeling.

This study demonstrates in airway smooth muscle (ASM) and normal human lung fibroblast cells (NHLF) that proliferation is potently modulated by isoprenoid-dependent signaling, particularly by GGPP. More importantly, data suggest that this pathway can be positively regulated by pharmacologically inducing elevated isoprenoid synthesis. Furthermore, leptin deficient ob/ob mice exhibit elevated RhoA in nasal epithelial tissue and tracheal smooth muscle cells. These data suggest that isoprenoid signaling is important in airway smooth muscle proliferation and viability and that this pathway could be a novel therapeutic target for treating obesity-related airway disease.

## 2. Materials and Methods

### 2.1. Cell Culture

 Human bronchial smooth muscle cells (BSMC) (Cambrex, Walkersville, MD) were cultured according to manufacturer instructions. All media, antibiotics, and growth factors were also supplied by Cambrex (Walkersville, MD) unless otherwise stated. Briefly, cells were seeded at a density of 3500 cells/cm^2^ in T-75 flasks in smooth muscle cell growth media supplemented with BulletKit 2 which includes 5% fetal bovine serum, 0.5 g/mL human recombinant epidermal growth factor, 5 mg/mL insulin, 1 g/mL human recombinant fibroblast growth factor, 50 mg/mL gentamicin, and 50 g/mL amphotericin-B. Media was replaced every 2-3 days until cells were 80% confluent. Cells were trypsinized according to manufacturer's instructions with 0.25 mg/mL trypsin/EDTA. Viable cells were counted using trypan blue exclusion and were plated per manufacturer's instructions in either 6-well dishes or 96-well microplates as appropriate.

 Normal human lung fibroblasts (NHLF) (Lonza, Walkersville, MD) were cultured according to manufacturer instructions. Briefly, cells were seeded at a density of 2500 cells/cm^2^ in T-75 flasks in fibroblast growth media BulletKit, which includes 5% fetal bovine serum, 1 g/mL human recombinant fibroblast growth factor, 50 mg/mL gentamicin, 50 g/mL amphotericin-B, and 5 mg/mL insulin. Media was replaced every 2-3 days until cells were 80% confluent. Cells were trypsinized according to manufacturer's instructions with 0.25 mg/mL trypsin/EDTA. Viable cells were counted using trypan blue exclusion and were plated per manufacturer's instructions in either 6-well dishes or 96-well microplates as appropriate. 

### 2.2. Proliferation Studies

Concentrations of mevastatin, GGPP, FPP, U18666a, and Y27632 were established in our previous studies [18–20]. Cells were treated with mevastatin at 0–20 *μ*M (Calbiochem, San Diego, CA) with or without geranylgeranyl pyrophosphate (GGPP) and farnesyl pyrophosphate (FPP) (Sigma, St. Louis, MO) at 10 *μ*M for 72 hours. Additional proliferation studies were done using GGPP and FPP alone at 10 *μ*M, U18666a (BioMol, Plymouth Meeting, PA) at 0.5 and 5 *μ*g/mL, and the Rho-associated kinase inhibitor Y-27632 (Calbiochem, San Diego, CA) at 0–50 *μ*M. The mevastatin and Y-27632 studies were done in basal smooth muscle or fibroblast media supplemented with BulletKit2. The studies examining the mitogenic potential of U18666a and FPP and GGPP were done in basal smooth muscle or fibroblast media supplemented with 5% FBS, 50 mg/mL gentamicin, and 50 g/mL amphotericin-B and 5 mg/mL insulin. Cells were plated in 96-well plates (5,000/well for ASM and 3,000/well for NHLF) in 100 *μ*L smooth muscle or fibroblast basal media with 0.5% serum and no growth factors for 24 hours prior to treatment. Proliferation was assessed by two methods, BrdU incorporation and MTS tetrazolium compound reduction. BrdU incorporation was assessed using a BrdU chemiluminescence kit purchased from Roche Applied Science according to manufacturer's instructions. After treatment, cells were labeled with BrdU for 24 hours and fixed and incubated with an anti-BrdU antibody conjugated with peroxidase. The immune complexes were measured by chemiluminescence detection in a luminometer. A blank control was performed in each assay according to manufacturer's instructions and was subtracted from the experimental groups. Results are reported as percent untreated controls (relative light units). Proliferation was also assayed by CellTiter 96 AQ_ueous_ One Solution Cell Proliferation Assay purchased from Promega (Madison, WI) and used per manufacturer's instructions. After treatment, 20 *μ*L CellTiter 96 AQ_ueous_ One Solution Reagent was added to each well in a sterile fashion and the plates were incubated for 2 hours at 37°C/5% CO_2_. Absorbance was read at 490 nm using a 96-well plate reader. The MTS tetrazolium salt in the reagent is bioreduced into a colored formazan product that is, proportional to the number of living cells in culture. Results are expressed as percent absorbance at 490 nm of untreated controls. Groups are compared using ANOVA with the Bonferroni correction. These studies were conducted to determine the role of isoprenoid signaling in cellular proliferation and were not intended to determine mechanisms of cell death.

### 2.3. Western Immunoblotting

 Western immunoblotting was performed as previously described [19]. Briefly, ASM and NHLF were seeded at densities of 3500/cm^2^ and 2500/cm^2^, respectively, in 6-well cell culture dishes and grown to 80–90% confluence at 37°C and 5% CO_2_. They were growth arrested in basal smooth muscle or fibroblast media supplemented with 0.5% FBS for 24 hours prior to experiments. The cells were treated with U18666a (5 *μ*g/mL in media supplemented with 5% FBS, 50 mg/mL gentamicin, 50 g/mL amphotericin-B, and 5 mg/mL insulin). Cells were lysed in 150 *μ*L cold lysis buffer (50 mM Tris, pH 7.5, 1% Triton X-100, 100 mM NaCl, 50 mM NaF, 200 *μ*M Na_3_Vo_4_, 10 *μ*g/mL pepstatin, and leupeptin (Sigma Chemical Co., St. Louis, MO)) for 30 minutes at 4°C. Plates were scraped to suspend cells, transferred to 1 mL microcentrifuge tubes, and centrifuged for 10 minutes at 14,000 rpm at 4°C. The supernatant was frozen at −20°C. Protein concentration of samples was measured using the Bio-Rad protein assay system (Bio-Rad Laboratories, Hercules, CA). Forty micrograms of protein were loaded and separated using sodium dodecyl sulfate/polyacrylamide gel electrophoresis (SDS/PAGE) through 7.5% acrylamide gel. Gels were transferred to Immobilon P membrane (Millipore, Bedford, MA). Blots were blocked in phosphate-buffered saline with 10% nonfat dry milk and 0.1% Tween-20 (Sigma Chemical Co., St. Louis, MO) overnight at 4°C. The blots were incubated with 1 : 1000 dilution of antibody to RhoA (Santa Cruz Biotechnologies, Santa Cruz, CA) at 4°C overnight and washed three times with PBS with 0.1% Tween-20. Secondary antibody conjugated to horseradish peroxidase was added (1 : 4000 dilution) for 1 hour at room temperature and washed three times with PBS with 0.1% Tween-20. Super Signal chemiluminescent substrate (Pierce, Rockford, IL) was added for 8–10 minutes at room temperature. The membranes were exposed to film (Kodak, Rochester, NY). Protein expression was quantified using Kodak Digital Science ID software and designated by mean pixel density. 

### 2.4. Mouse Nasal Epithelium and Tracheal Cells

 8-week-old ob/ob leptin knockout mice were compared to age- and background-matched control mice. Mice were originally obtained from the Jackson Laboratory, with a breeding colony maintained in the Animal Research Center at Case Western Reserve University. All mice were fed a regular diet (irradiated Prolab RMH 3000) and sterile water. Mice were housed in static isolator units on corncob bedding. All mice were cared for in accordance with Case Western Reserve University Internal Animal Care and Use Committee guidelines. Mice were sacrificed by carbon dioxide narcosis, and the nasal epithelium was extracted. Excised nasal epithelial tissue was placed in 1X Passive Lysis Buffer (Promega, Madison, WI) and then centrifuged to obtain protein extract (supernatant). After the nasal epithelium was excised, the trachea lungs were excised. Tracheal smooth muscle cells were harvested as previously described [28]. The trachea was dissected from the larynx to the proximal right and left mainstem bronchi. Surrounding connective tissue was removed, and the tracheas were minced and digested in collagenase, elastase, and soybean trypsin inhibitor, filtered, and washed in Hanks' buffered saline. Cells were seeded into flasks in T-75 flasks in smooth muscle cell growth media BulletKit 2. Cells were passaged between days 5 and 8 when 80–90% confluent. Cells were characterized as >85% smooth muscle cells by indirect immunostaining for smooth muscle actin (Sigma, St. Louis, MO). Primary, first-, and second-passage cells were used for the studies.

## 3. Results

### 3.1. RhoA Expression in ob/ob Mouse Nasal Epithelium and Tracheal Smooth Muscle Cells

**Figure 2:**
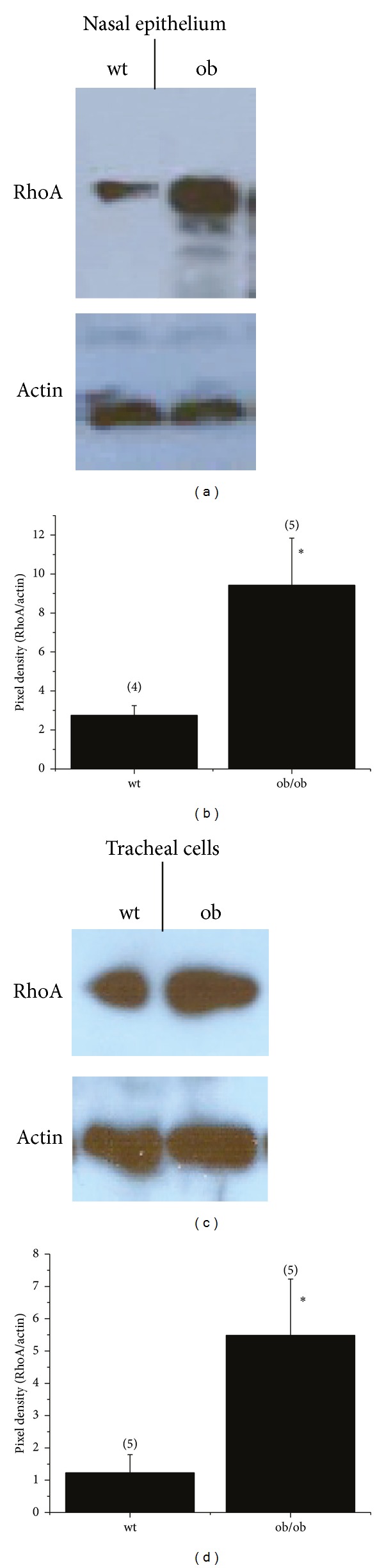
RhoA expression is increased in obese mice. RhoA expression in mouse nasal epithelium and tracheal smooth muscle cells from wild-type (wt) and leptin-deficient (ob) mice. (a) Representative gel showing RhoA and actin expression from excised mouse nasal epithelium. (b) Relative density (RhoA/actin) quantification of RhoA protein content in nasal epithelium of wt and ob/ob mice. (c) Representative gel showing RhoA and active expression from mouse tracheal smooth muscle cells. (d) Relative density (RhoA/actin) quantification of RhoA content in tracheal cells from wt and ob/ob mice. Error bars represent SEM; *P = 0.05. Number of replicates is shown in parentheses above each bar. Replicates represent individual mice.

 Leptin-deficient ob/ob mice exhibit elevated expression of GGPS [26] as well as increases in inflammation and airway hyperresponsiveness [9–14]. Based on our previous work [18, 19], it is hypothesized that ob/ob mice would exhibit an increase in RhoA expression just as we observed in CF and NPC models. RhoA expression was examined initially in mouse nasal epithelium (MNE), a useful model of airway signaling [29], and tracheal smooth muscle cells. MNE and tracheal smooth muscle cells from ob/ob mice exhibit significantly elevated RhoA expression compared to wt controls (Figure 2). RhoA expression is increased in CF and NPC presumably due to cholesterol processing changes that increase isoprenoid/cholesterol synthesis [18–20]. Although we do not explore the direct cause of RhoA expression increases in the obese mouse model, we hypothesize that isoprenoid signaling may impact airway smooth muscle and fibroblast viability and proliferation as is seen in vascular smooth muscle.

### 3.2. Mevastatin Inhibits ASM and Normal Human Lung Fibroblast (NHLF) Growth

**Figure 3:**
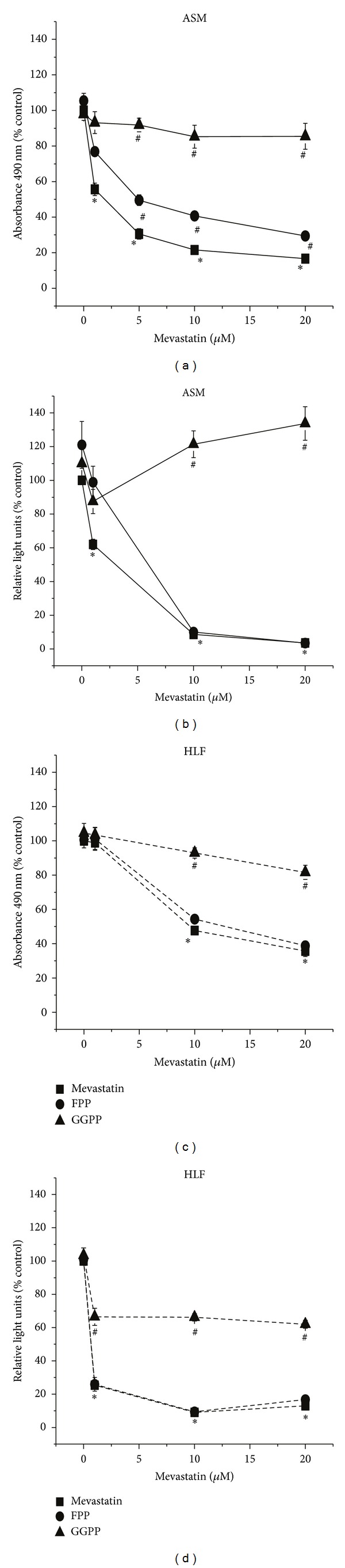
Mevastatin inhibits ASM and NHLF proliferation. Airway smooth muscle cell (ASM, shown as solid lines) and normal human lung fibroblast (NHLF shown as dotted lines) proliferation are decreased in a dose-dependent fashion by mevastatin (■). This effect is reduced somewhat by FPP (●) and is almost abolished by GGPP (▲), suggesting specificity to the Rho family of GTPases. ASM cell proliferation assayed by (a) MTS tetrazolium compound reduction (CellTiter, Promega) and (b) BrdU incorporation. HLF fibroblast proliferation assayed by (c) MTS tetrazolium compound reduction and (d) BrdU incorporation. Data shown are the mean (±SE) of three experiments, each performed in triplicate. Groups are compared by ANOVA with the Bonferroni test; *P < 0.05 compared to untreated, ^#^ *P* < 0.05 compared to mevastatin treatment alone.

 One hypothesis of this study is that if ASM cell growth is dependent on isoprenoid signaling, then the inhibition of isoprenoid synthesis with mevastatin would result in growth inhibition and cell death as is observed in VSM. As hypothesized, a dose-dependent reduction of ASM cell viability is seen after treatment for 72 hours with mevastatin (0–20 *μ*M) resulting in a 83.4 ± 0.7% reduction in cell viability as measured by MTS tetrazolium salt reduction at the 20 *μ*M concentration (Figure 3(a)). In order to determine if mevastatin sensitivity is due to isoprenoid-dependent signaling, experiments were performed in the presence of either FPP (10 *μ*M) or GGPP (10 *μ*M). Mevastatin-mediated cell number reduction was reduced to 44.4 ± 3.5% in the presence of FPP, and cells were completely protected in the presence of GGPP (Figure 2(a)). To more directly measure mechanisms of proliferation, the same experiment was performed measuring BrdU incorporation. The highest concentration of mevastatin (20 *μ*M) reduced BrdU incorporation by 96.5 ± 0.8% in ASM (Figure 3(b)). The addition of FPP had no influence on mevastatin-mediated inhibition of BrdU incorporation, while GGPP addition completely restored BrdU incorporation in the presence of mevastatin (Figure 3(b)). These data clearly demonstrate that a GGPP-specific pathway is critical to maintaining ASM cell growth.

Statin therapy has also been implicated as a protective agent in the development of pulmonary fibrosis [29]. Therefore, we examined whether growth regulations of ASM and NHLFs were regulated similarly by isoprenoid-dependent pathways. Similar findings with respect to NHLF are shown in Figures 3(c) and 3(d). The highest concentration of mevastatin (20 *μ*M) inhibited growth of NHLF compared to untreated controls by 64.3 ± 3.2% as measured by MTS tetrazolium salt reduction. BrdU incorporation was also in a dose-dependent manner by mevastatin in NHLF, with a maximum inhibition of 90.9 ± 0.6% at 20 *μ*M. As with ASM cells, the effect of mevastatin was reversed in NHFLs by the addition of GGPP, but not by the addition of FPP. These results demonstrate that inhibiting isoprenoid synthesis decreases ASM and NHLF viability but do not speak to whether the mechanism of cell death is apoptosis or necrosis.

### 3.3. Potential Role of RhoA-Mediated Signaling in ASM and NHLF Growth Regulation

**Figure 4:**
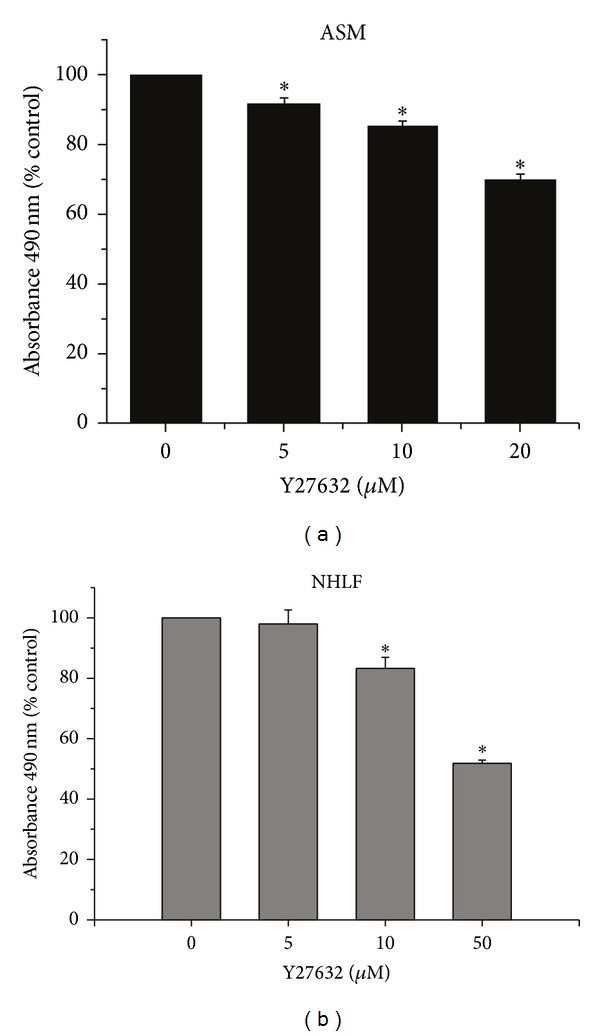
*Inhibiting ROCK decreases ASM and NHLF proliferation. *Inhibiting the Rho-associated kinase (ROCK), which mediates some of the downstream effects of RhoA, reduces (a) ASM (black bars) and (b) NHLF (grey bars) proliferation, assayed by MTS tetrazolium compound reduction (CellTiter). Data shown are the mean (±SE) of *n* = 36, run in 3 experiments (12 replicates each experiment); groups are compared by ANOVA with the Bonferroni test, *P < 0.05.

 The dependence on GGPP in regulating cell growth suggests the possible involvement of the Rho family of small GTPases, particularly RhoA in light of previous studies on vascular smooth muscle regulation [25, 30, 31]. To assess the potential role of RhoA signaling in this process, we examined the effect of the Rho-associated kinase (ROCK) inhibitor Y27632 on ASM and NHLF cell growth. ROCK mediates many of the downstream effects of RhoA [32]. Y-27632 reduced ASM and NHLF cell viability as measured by MTS tetrazolium salt reduction, although responses were less robust than with mevastatin (Figure 4). ASM cell viability was reduced by 8.5 ± 1.8% to 30.1 ± 1.8% at doses of 5 and 20 *μ*M, respectively (Figure 3(a)). Similar results were seen in NHLF (Figure 3(b)) with 16.7 ± 3.6% and 48.2% reduction in absorbance compared to untreated controls at doses of 10 to 20 *μ*M Y-27632, respectively. Not all functions of RhoA are mediated by ROCK activation, which might explain the reduced effect of ROCK inhibition. Direct inhibition of RhoA is challenging, and introducing dominant-negative RhoA (dn-RhoA) constructs into these cell lines proved difficult. Although these results do not directly confirm an involvement of RhoA in smooth muscle growth regulation, they are consistent with the specificity of GGPP in this pathway and further point to an important role for RhoA as a mediator of signaling in ASM. 

### 3.4. Positive Regulation of Isoprenoid Synthesis Increases ASM Proliferation and RhoA Expression

 Inhibition of ASM growth via intervention in the isoprenoid/RhoA pathway as shown above is expected based on comparison with VSM studies, and these results confirm those recently reported by Takeda et al. [33]. The next goal was to determine if elevating isoprenoid synthesis would lead to accelerated ASM and NHLF proliferation. 

**Figure 5:**
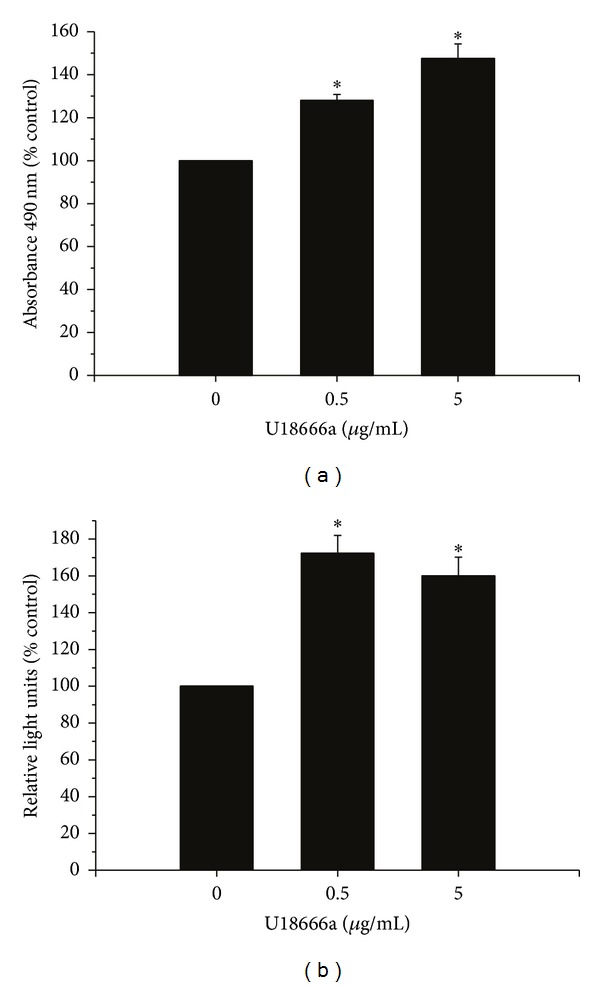
Upregulating isoprenoid production increases ASM viability and proliferation. Increasing isoprenoid productionusing U18666a increases ASM proliferation as assayed by (a) MTS tetrazolium compound reduction (CellTiter, Promega) and (b) BrdU incorporation. Data shown are the mean (±SE) of *n* = 36, run in 3 experiments (12 replicates each experiment); groups are compared with ANOVA with the Bonferroni test, *P < 0.05 compared to no treatment (NT).

Demonstrating positive regulation of cellular proliferation by upregulating isoprenoid-mediated signaling in normal ASM is a necessary first step in supporting the contention that obesity affects airway disease through increases in this pathway. To accomplish this goal, cells were treated with the compound U18666a, an inhibitor of intracellular cholesterol transport [34]. U18666a (3-beta-[2-(diethylamino) ethoxy]androst-5-en-17-one) is a class 2 amphiphile known to inhibit intracellular cholesterol transport and leads to NPC-like free cholesterol accumulation. Although U18666a actually inhibits cholesterol synthesis, it has been demonstrated to elevate isoprenoid production as measured by ubiquinone production [35]. ASM proliferation was increased in the presence of the maximum concentration of U18666a (5 *μ*g/mL) by 147.6 ± 6.7% assayed by MTS tetrazolium salt compound reduction (Figure 5(a)). BrdU incorporation was increased in ASM cells by 172.4 ± 9.6% in the presence of U18666a (5 *μ*g/mL) (Figure 5(b)). 

**Figure 6:**
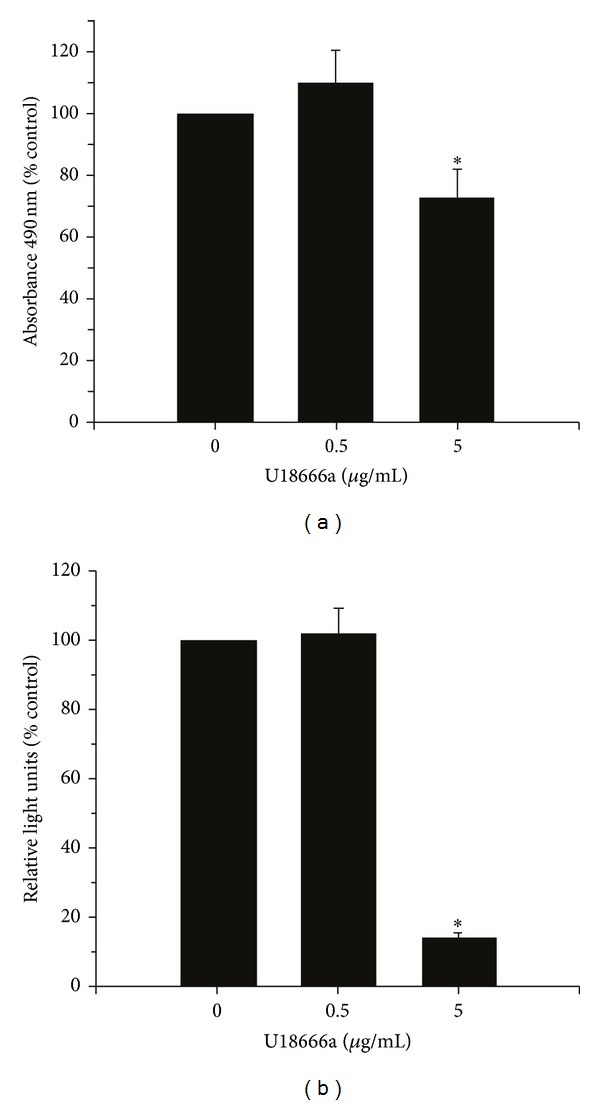
U18666a decreases NHLF viability. Increasing isoprenoid production and inhibiting cholesterol transport with U18666a inhibit the growth of normal human lung fibroblasts, assayed by (a) MTS tetrazolium compound reduction (CellTiter) and (b) BrdU incorporation. Data are shown as the mean (±SE) of *n* = 36 run in 3 experiments (12 replicates each experiment); groups are compared by ANOVA with the Bonferroni test, *P < 0.05.

 The effect of U18666a treatment was also assessed on NHLFs since fibroblasts exhibited similar results to ASM with respect to mevastatin treatment. Interestingly, fibroblast proliferation was inhibited by U18666a (5 *μ*g/mL) using both MTS tetrazolium salt compound reduction, and BrdU incorporation as measures (Figures 6(a) and 6(b)). Cell viability was decreased by 27.4 ± 9.3% assayed by MTS tetrazolium salt compound reduction and BrdU incorporation was reduced by 85.6 ± 1.2%. This finding is more consistent with previous reports of the effect of U18666a on neuronal cells where apoptosis is induced [36, 37]. Thus, the increase in ASM proliferation observed in this study following stimulation of isoprenoid is contradictory to other cell lines including fibroblasts and suggestive of an airway specific response.

**Figure 7:**
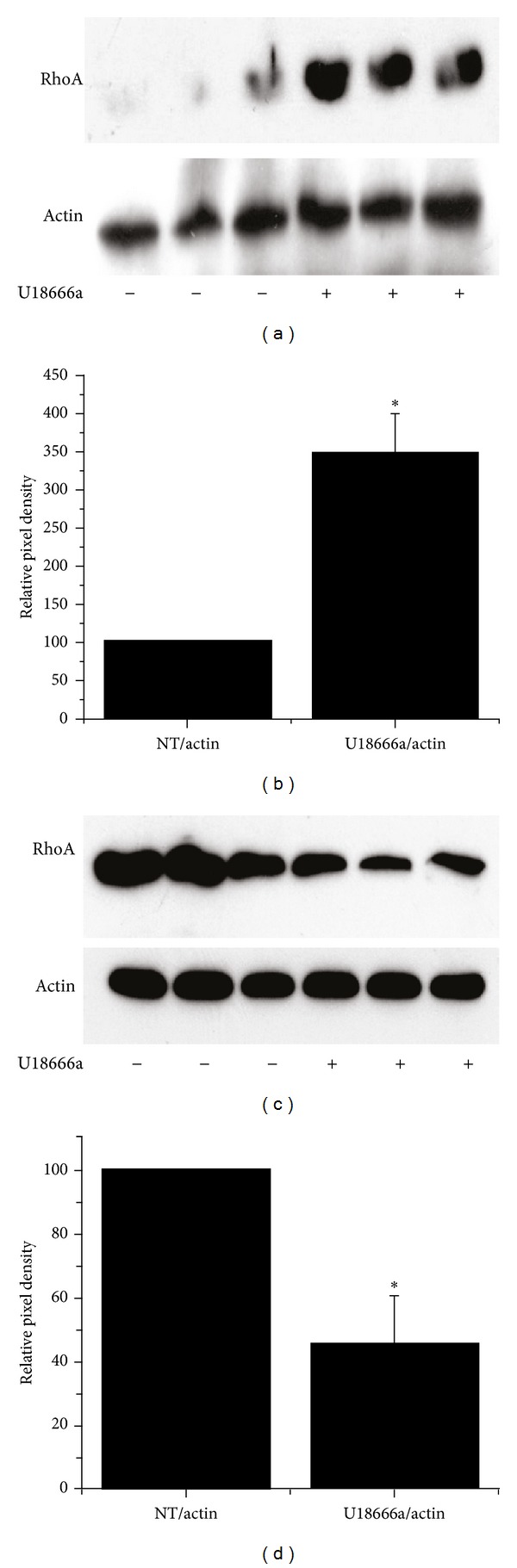
U18666a increases RhoA expression in ASM and decreases RhoA expression in NHLF. RhoA expression by western blot is increased in ASM after 72 hours of treatment by U18666a (5 *μ*g/mL) compared to untreated cells (NT) but is decreased in NHLF cells, which is consistent with the differential effects of U18666a on cell proliferation in these cell types. (a) Representative blot of ASM. (b) Densitometry shown as mean of 3 experiments, each run in duplicate or triplicate; RhoA density is normalized to actin. (c) Representative blot of NHLF. (d) Densitometry shown as mean of 3 experiments, each run in duplicate or triplicate; RhoA density is normalized to actin. Means are compared by the Student's *t*-test, *P < 0.05.

 Although both FPP and GGPP influence U18666a-mediated increases in ASM proliferation, our hypothesis is that endogenous ASM cell growth is dependent primarily on geranylgeranyl-mediated signaling likely through RhoA. To determine if differential effects of U18666a treatment on cell growth between NHLF and ASM could be due to differential regulation of RhoA, RhoA protein expression was examined in NHLF and ASM cells after treatment with U18666a. Figures 7(a) and 7(c) show representative blots demonstrating elevated RhoA protein content in ASM and reduced RhoA protein content in NHLFs subsequent to treatment with U18666a. ASM cells exhibit a 3.5 ± 0.5 fold increase of RhoA protein as quantified by densitometry after being treated with U18666a (5.0 *μ*g/mL) (Figure 7(b)). However, NHLF samples exhibit a 0.5 ± 0.2 fold decrease in RhoA expression after U18666a treatment (Figure 7(d)).

## 4. Discussion

We made the observation that RhoA expression is significantly increased in airway tissue (tracheal smooth muscle and excised nasal epithelium) from ob/ob mice compared to wt controls. This finding is consistent with other findings in our laboratory that demonstrate increased RhoA expression and activation in both CF and NPC samples [18–20]. Both CF and NPC have in common the phenotype of increased cholesterol synthesis due to perinuclear-free cholesterol accumulation [21, 22]. The mechanisms of how obesity leads to RhoA expression is currently under investigation; however, our previous work points to a potentially important role of isoprenoid signaling in airway regulation. We have demonstrated that NPC skin fibroblasts exhibit heightened sensitivity to isoprenoid inhibitors and that CF cells exhibit isoprenoid-dependent signaling control [19, 20]. The increase of RhoA in obese mouse airway tissue led us to explore the question of whether airway smooth muscle growth and viability were inherently sensitive to isoprenoid pathways as has been shown in vascular smooth muscle. Such a finding would suggest that obesity-driven airway disease could be treated with statins as an easily accessible inhibitor of isoprenoid synthesis. 

Considerable attention is being focused on the benefits of statin therapy independent of its ability to lower plasma cholesterol, with a focus on the consequences of interfering with isoprenylation. Specific benefits of statin therapy include the inhibition of vascular smooth muscle contraction via the disruption of the RhoA signaling pathway [38]. The RhoA pathway has also been implicated in the regulation of vascular smooth muscle proliferation, and statin-induced smooth muscle apoptosis has been postulated to be an early benefit of statin therapy [39] in addition to a number of other benefits. 

Our data demonstrate that ASM and NHLF growth are indeed regulated by the isoprenoid/RhoA pathway confirming results recently reported by Takeda et al. [33]. These data established that fibroblast and muscle growth regulation can be isoprenoid/RhoA dependent and easily inhibited with modulators of these pathways. Further work will be needed to define the mechanism of growth inhibition. However, another goal was to determine if ASM and NHLF proliferation could be positively regulated by increasing the isoprenoid synthesis pathway pharmacologically. This study demonstrates that ASM proliferation is accelerated by increasing isoprenoid production, while NHLF growth is actually retarded, findings that correspond to RhoA expression. The observed positive growth regulation suggests that physiological conditions that alter RhoA activation or isoprenoid production could lead to adverse pulmonary health. 

The role of isoprenoid mediated signaling in airway smooth muscle proliferation may represent a unique therapeutic target, particularly in obese patients with airway disease. Epidemiologic data link obesity and the development of asthma in some populations [4–6, 8], but the pathophysiologic explanation for this association is not well understood. Goalstone et al. have provided evidence of elevated isoprenoid-related signaling in ob/ob mouse liver, aorta, and skeletal muscle by demonstrating elevated content of farnesylated Ras GTPase [27]. Lui et al. [40] reported elevated activity of Rho kinase, a downstream mediator of RhoA, in obese subjects with metabolic syndrome. Similarly, Matsumoto et al. reported increased RhoA activation and increased mesenteric artery contraction in ob/ob in response to 5-hydroxytyrosine [41]. These findings are consistent with our observation of increased RhoA content in airway smooth muscle and epithelial tissues from a mouse model of obesity. 

In conclusion, this paper demonstrates that nasal epithelial and tracheal smooth muscle tissue from ob/ob mice exhibit an increase in RhoA expression, and that airway smooth muscle cells and lung fibroblasts viability and proliferation are influenced by isoprenoid-dependent and RhoA/ROCK signaling. The mechanistic source of increased RhoA expression in airway tissues of obese mice needs to be determined, as does the specific impact of this pathway on the airway pathophysiology of obesity. However, the key role of isoprenoid/RhoA signaling in regulating ASM and NHLF viability and proliferation suggests that this pathway is an intriguing target for treatment of obese asthmatics.
